# Perspective on Cancer Therapeutics Utilizing Analysis of Circulating Tumor Cells

**DOI:** 10.3390/diagnostics8020023

**Published:** 2018-04-11

**Authors:** Keun-Yeong Jeong, Eun Kyung Kim, Min Hee Park, Hwan Mook Kim

**Affiliations:** 1 R&D Division, Metimedi Pharmaceuticals Co., 263, Central-ro, Yeonsu-Gu, Incheon 22006, Korea; oncometeun@gmail.com (E.K.K.); pmh1880@hanmial.net (M.H.P.); 2 Gachon Institute of Pharmaceutical Science, Gachon University, 191, Hambangmoe-ro, Yeonsu-gu, Incheon 21936, Korea

**Keywords:** solid cancer, circulating tumor cells, cancer screening, cancer diagnosis, cancer therapeutics

## Abstract

Various methods are available for cancer screening, and the methods are performed depending on the origin site of cancer. Among these methods, biopsy followed by medical imaging is the most common. After cancer progression is determined, an optimal treatment—such as surgery, chemotherapy, and/or radiation therapy—is selected. A new assay has been developed that detects circulating tumor cells (CTCs). Tracking changes in CTCs may reveal important tumoral sensitivity information or resistance patterns to specific regimens and prompt changes in therapy on a personalized basis. Characterization of CTCs at the DNA, RNA, and protein levels is important for gaining insight for clinical applications. A small number of CTCs can be analyzed to obtain genome information such as the progression of cancer including metastasis, even in a single cluster. Although many clinical studies, particularly CTC enumeration and detection of specific oncogene expression, have increased the success rate of diagnosis and predicting prognosis, there is no consensus regarding the technical approaches and various aspects of the methodology, making it difficult to standardize optimal methods for CTC analysis. However, ongoing technological advances are currently being achieved and large-scale clinical studies are being conducted. Applying CTC analysis in the clinic would be very useful for advancing diagnosis, prognosis prediction, and therapeutics.

## 1. Cancer Screening and Diagnosis

Cancer screening aims to detect cancer in order to achieve diagnosis before cancer progression. Well-known methods for screening include blood, urine, biopsy, or medical imaging tests. If cancer-related signs are detected, more conclusive and invasive methods are performed to confirm the diagnosis. Therefore, different methods for cancer screening are performed depending on the site. Screening involves evaluating the origin site before signs or symptoms of the cancer appear. According to the guidelines of the United States Preventive Services Task Force (USPSTF), regular mammograms can lower the risk of breast cancer-related death (https://www.uspreventiveservicestaskforce.org). A mammogram is a low-dose X-ray that detects changes in breast tissue. Another imaging method for screening and diagnosis of breast cancer is magnetic resonance imaging (MRI), which is a non-invasive imaging technology that produces three-dimensional detailed anatomical images [1]. MRI is conducted along with mammograms to screen for high-risk breast cancer [1,2]. The USPSTF recommends screening for colorectal cancer using fecal occult blood testing, sigmoidoscopy, or colonoscopy in adults [3]. A recent screening test based on the detection of the enzyme M2-pyruvate kinase (an oncoprotein secreted by colorectal cancers and detectable in stools) was developed to detect colorectal cancer and polyps [4]. Additionally, Cologuard^®^ has been established as a noninvasive screening test for colorectal cancer that analyzes stool DNA [5]. In the prostate cancer screening test, prostate-specific antigen (PSA) is detected, and this screening has been shown to help prevent prostate cancer-related deaths [6]. However, current methods for analyzing PSA result in overdiagnosis and various side effects, such as bleeding and infection [7,8]. Therefore, the USPSTF recommends that diagnosis by PSA alone is not sufficient. Additionally, endoscopic ultrasound and MRI/computed tomography imaging can be utilized for cancer screening and diagnosis, including pancreatic cancer for which symptoms are difficult to detect [9]. Although multiple screening and diagnostic methods of cancers provide treatment opportunities through early detection, screening can lead to false-positive and false-negative results, involve invasive procedures, or existing cancer is missed. Therefore, several factors are considered in determining whether the benefits of screening outweigh the risks and costs. Some types of screening tests, such as X-ray imaging, expose the body to potentially harmful ionizing radiation [10]. There is a small chance that the radiation in the test can cause a new cancer in a healthy person. Screening mammography is not recommended for men or young women because they are more likely to be harmed by the test than to benefit from it [11]. Further, if the test is not specific, an incorrect cancer diagnosis may be made in a healthy person [11,12]. If the screening test is positive, further diagnostic testing is typically conducted, such as tissue biopsy; however, if the test produces many false-positives, many people will undergo needless medical procedures, some of which may be dangerous [12]. To reduce these risks, the development of personalized medicine for patients with cancer depends on the identification of the molecular drivers of their disease. Biomarkers for predicting treatment response are most frequently measured in tumor biopsy samples. However, given that cancer is continually evolving at the molecular level, whether a diagnostic biopsy sample truly represents the patient’s disease is questionable. To overcome these issues, a new assay has been developed to detect circulating tumor cells (CTCs) and showed clinical significance in cancer diagnosis. This assay may play an essential role in determining effective therapeutics.

## 2. Cancer Therapeutics and Circulating Tumor Cells (CTCs)

Currently, various chemotherapies have been developed based on advanced knowledge. Many studies have identified targets for the application of new drugs. In addition to antibodies and inhibitors, researchers are developing novel classes of molecules targeting signaling cascades and specific gene mutations in cancer cells, known as target drugs. Furthermore, recent studies identified several proteins on the surface of T cells that function as checkpoints. Cytotoxic T-lymphocyte-associated antigen 4 and programmed cell death protein-1 are checkpoints in immune cells that may be turned off by chemotherapy [13,14]. Viruses were also found to be effective for manipulating malignancies in humans. Particularly, adenoviruses have been widely studied in humans, resulting in the development of gene therapy [15,16]. Gene therapy aims to replace abnormal genes in cancer cells, with most approaches addressing critical gene defects [15]. One way to improve the pharmacodynamics of cancer treatments is by using nanotechnology. Nanotechnology can be used to target chemotherapies directly and selectively to neoplasms. It also enhances the therapeutic efficacy of current treatment modalities [17]. Additionally, robotic surgery has attracted attention as a potential therapy for cancers. Robotic systems have been used for several types of cancer surgery as mechanical and computer technology has improved, and the system can remove tumors more completely and with less surgical trauma than conventional techniques [18]. However, these therapeutics cannot be immediately applied to individual patients; accurate diagnosis of cancer must depend on the site of the cancer and appropriate therapy is selected after detailed investigation. Tracking changes in CTC values over time and during therapy may reveal important tumoral sensitivity or resistance patterns to specific regimens and prompt changes in therapy on a personalized basis [19,20]. CTC analysis may be performed on isolated tumor cells to alter a patient’s chemotherapy course without requiring multiple invasive procedures [19]. CTCs originate from a primary tumor and are dispersed in the peripheral circulation among millions of immune cells and red blood cells [19,21]. Detection of these CTCs is revolutionizing the understanding of the pathogenesis of metastasis and providing a foundation for sensitive techniques to detect malignancy, monitor recurrence, and prognosticate outcomes [19]. In recent years, numerous procedures and platforms have been developed to isolate CTCs for further molecular biological analyses. The emergence of these technologies has spurred great interest in CTCs, and many clinicians are realizing the importance of CTCs in cancer biology as well as their use in cancer therapeutics.

## 3. Concept for Circulating Tumor Cells (CTCs) Analysis

Most cells circulating in the blood in a patient with a cancer, except for leukemia, are normal blood cells, specifically white blood cells. Thus, the relatively small number of CTCs is difficult to detect against this large background of normal blood cells. The simple concept of CTC analysis is to gather or detect small numbers of CTCs from among larger numbers of white blood cells. Existing techniques for CTC isolation can be divided into three types: antibody-, physical property-, and function-based approaches [22,23,24]. A recently established microfluidic method is based on CTC isolation techniques that divided into three types as above. Cell surface proteins are often used as targets in antibody-based CTC isolation methods. Because of the lack of a strictly tumor-specific antigen, epithelial-specific proteins—such as epithelial cell adhesion molecule (EpCAM)—are used for CTC-positive selection and antibodies against leukocyte-specific surface antigens, such as CD45, are used to deplete leukocytes [24,25]. Technologies based on physical properties dominate the field of CTC separation. Synthetic low-viscosity high-molecular weight hydrophilic sucrose, epichlorohydrin polymer, and sodium diatrizoate are widely used to deplete erythrocytes by density gradient centrifugation [22]. Another strategy for enriching viable CTCs is to acquire a specific number of CTCs based on functions. Methods detecting the ability of tumor cells to attach and ingest collagen adhesive matrices have been described as collagen adhesive matrix assays [23,26]. Benign or normal epithelial cells do not circulate in the blood. In contrast, CTCs are derived from the primary site of solid cancers such as colon, lung, and breast cancer for metastasis [27]. Representative techniques are available for analyzing rare tumor cell populations such as CTCs, which can be subdivided into DNA, RNA, protein, glycosylation-based methods.

### 3.1. DNA-Based Genome Analysis

The characterization of CTCs at the DNA level has potential to provide biological insight and clinical applications. For example, in colorectal cancer, the Ras and Raf mutations in a specific genome, such as in exon 2, show a pattern that has been established as a negative marker for tyrosine kinase inhibitors [28]. Thus, monitoring of CTC-derived genotypes may provide a noninvasive approach for identifying drug sensitivity and optimal therapeutic decisions. The analysis of single or cluster CTC genomes can be analyzed using well-known methods or recent technologies simultaneously. Interphase cytogenetics, utilizing fluorescence in situ hybridization techniques, has been successfully applied to analyze CTCs derived from solid cancer [29]. This method was originally useful to detect specific chromosomal translocations, inversions, or deletions in both metaphase and interphase. Whole genome amplification (WGA) is a method for robust amplification of an entire genome using nanogram quantities of DNA from a single CTC [29]. This amplification is aimed to increase DNA amounts from those of one CTC to yields sufficient for different DNA analyses for next-generation sequencing or array-comparative genome hybridization [30]. Two techniques for the amplification of genomic DNA from fixed single CTCs isolated from blood of cancer patients are described. These techniques are advanced polymerase chain reaction (PCR), such as a linker-adaptor PCR and improved primer extension preamplification PCR, and multiple-strand displacement amplification. In addition, several commercial kits for WGA have been developed [29,30]. The CTC whole-genome amplification product can then be analyzed for copy number changes by array-comparative genome hybridization [30]. Furthermore, next-generation sequencing offers the opportunity to establish the mutation spectrum of multiple genes. Therefore, genome analysis in CTCs can be used for quantification and molecular characterization of carcinoma cells and for monitoring the response to anticancer therapy [30,31].

### 3.2. RNA-Based Gene Expression Analysis

CTC-based gene expression monitoring represents a new direction in the development of a precision medicine approach tailored to provide information on the specific progression step of the cancer. Gene expression profile analysis is carried out on CTCs quantified from the blood of advanced cancer patients, providing information regarding cancer progression and metastasis [32]. For example, co-expression of genes that code for the epithelial marker EpCAM and mesenchymal markers fibronectin and matrix metalloproteinases in CTCs of cancer patients suggests a partial epithelial to mesenchymal transition (EMT) [24,33]. If partial EMT is detected in the analysis, it can be predicted that tissue fibrosis has been initiated or deteriorated in the primary site of cancer patients, which is correlated with poor prognosis because of tumor progression and metastasis [33]. Furthermore, initiation of bone metastasis from primary cancer can be identified by analyzing CTC gene expression; increased expression of the bone morphogenetic protein-7 gene is a useful marker and member of the transforming growth factor-beta family that is typically expressed in osteoblastic bone metastases [34]. As described above, gene expression analysis is different from genome analysis because it confirms a mutation in CTCs and oncogene expression related to cancer progression and metastasis in CTCs [32]. To perform gene expression analysis, an RNA amplification process, such as WGA for genome analysis, is required. Whole-transcriptome amplification (WTA) is a simple amplification protocol that is analogous to WGA [35]. In WTA, RNA undergoes a single-step conversion into cDNA fragments using universal priming sites, and PCR amplification is subsequently performed using universal oligonucleotide primers [35]. cDNAs amplified from the RNA are commonly used for PCR analysis using an oncogene-based primer pair. Further, RNA-based expression monitoring with and without WTA is conducted in CTC-enriched cells [33,35]. Recently, using cluster CTCs from the blood, single-molecule RNA sequencing revealed upregulation of *WNT* genes, which was associated with distal spread in cancer. For example, RNA-fluorescence in situ hybridization to examine expression of seven pooled epithelial transcripts such as EpCAM, fibronectin 1, and cadherin 2 supported the contribution of EMT in CTCs of patients with cancer [24,36,37]. Single-molecule RNA sequencing, which is based on real-time PCR, and direct analysis using RNA expression array can also be used to analyze oncogene expression in CTCs [30,35]. These single-molecule sequencing methods enable RNA to be sequenced directly from biological samples, making it well-suited for diagnostics in the clinic. Therefore, RNA-based gene expression analysis in CTCs will contribute to establishing whether or not cells that fulfill the “CTC criteria” with current capturing systems are indeed tumor cells [31].

### 3.3. Protein-Based Analysis

Viable CTCs can be analyzed by detecting secreted proteins such as specific antigens or cytokeratins by the epithelial immunospot assay [19,38]. The protein analysis tool may model the evolution of metastatic cancer and, importantly, be used for drug testing. Isolated CTCs are then cultured in a tissue culture plate pre-coated with antibodies that capture the secreted protein. The secreted proteins are therefore immobilized around the CTCs which produce these proteins and forms spot [37,38]. After the incubation period, the cells are removed, and secreted proteins are detected by immunological techniques and counted [38]. In experiments examining cancer cell lines, the method can detect one cancer cell in approximately 5 mL of blood [19]. Additionally, cathepsin D or cell surface-associated mucin 1 (*MUC*1)-secreting cells were successfully detected in cancer patients. Elevated levels of cathepsin D indicate the recurrence of breast cancer. Overexpression of cathepsin D is also associated with tumorigenesis of colorectal cancer (CRC) and lung adenocarcinoma and with aggressive forms in prostate cancer [39]. *MUC*1 is a transmembrane mucin that acts as a tumor-associated antigen in various carcinomas including metastatic progression [39,40]. Furthermore, cytokeratin 19, an intermediate filament protein involved in the structural integrity of epithelial cells, was detected in up to 65% and 70% of CRC and breast cancer patients, respectively [39,41]. Therefore, it is possible to predict poor prognosis and correlate the results with the presence of metastasis and poor survival in patients with cancer [31].

### 3.4. Glycosylation-Based CTC Analysis

Alterations in glycosylation structures in CTCs could serve as important diagnostic markers [42]. Malignancy condition in cancer, such as hypoxia, has been identified as one of the factors leading to increased expression of glycosyltransferases. For instance, increased expression of 1,3-fucosyltransferase-7 and α2,3-sialyltransferase enzymes is associated with a metastatic cell phenotype. Therefore, These appearance could be related to prediction of poor outcome, independent of tumor size and grading at diagnosis [42]. The analysis of glycosylation of CTCs can be analyzed by carbohydrate microarrays and a high-speed fiber-optic array scanning technology. Arrays of glycans with micro arrays are used for the high throughput mapping of cell wall polysaccharide populations across a range of samples [43,44]. Using a carbohydrate microarray, the anti-tumor monoclonal antibody is tested against a large panel of carbohydrate antigens and a potential tumor glycan marker can discover in CTCs [43]. Flow cytometry and optical fiber array scanning techniques are then applied to determine whether they are the identified target tumor-specific glycan markers [45]. In another application, the tumor glycan-specific antibodies can validate using blood samples of patients with cancer for their performance in CTC-detection [46]. However, because there are variety of glycosylation types and structure, a systematic study of representative glycan markers along with various types of cancer and progressive stages is to be useful for physicians.

### 3.5. Functional Analysis with CTC Culture

Isolation of viable CTCs is technically challenging because most methods yield low numbers of partially purified CTCs that are damaged during the cell purification process [21]. The limited number of CTCs limit the ability to perform comprehensive studies, particularly functional studies. Therefore, the proliferation of CTCs is essential for detailed functional analysis. An advanced culture technology using successfully isolated-CTCs provides various opportunities to utilize CTCs for diagnosis and prognosis prediction [37,38]. Two-dimensional (2D) culture, three-dimensional (3D) culture, and xenotransplantation are the main proliferation strategies [47,48]. Among them, standard 2D cell culture conditions are widely used, particularly for short-term culture. 2D culture of CTCs can facilitate single-cell analysis and gene expression analysis for CTCs [47]. As examples of short-term culture and analysis, breast cancer-derived cytokeratin 19 protein-releasing CTCs are associated with metastasis and can predict low overall survival [39]. However, for primary 2D culture, specific medium or feeder cells should be used, and the success rate are low under the non-adherent conditions of CTCs in long-term cultures [48]. Adherent conditions are important for long-term production of viable of CTCs. To overcome this problem, a 3D culture method was applied for long-term proliferation of CTCs. To form 3D conditions, extracellular matrix, often referred to as organotypic culture or organoid culture, is often utilized [48]. Xenotransplantation is another strategy for proliferating CTCs and is quite different from 2D and 3D culture systems [47,48]. 3D cultures using xenotransplantation have great potential for developing personalized cancer medicine such as for treatment efficacy testing. Therefore, culture of CTCs in the blood of cancer patients can be conducted to study patterns of drug susceptibility, linked to the genetic context unique to individual tumors. However, before incorporating this strategy into clinical practice, optimization of culture conditions is necessary. In addition, nonadherent CTC-derived cell lines should be further characterized to determine how they differ from cells cultured with a primary tumor or cells directly transplanted into a mouse model [31].

## 4. Clinical Application of Circulating Tumor Cells (CTCs) Analysis

There is a clear need for improved cancer biomarkers in the clinic, and CTCs have been proposed as a very effective solution. This is because CTCs have numerous very desirable characteristics as cancer biomarkers. CTCs are have been detected in the peripheral blood of the most common solid tumors, suggesting that CTCs can be universally applied in cancer screening [19,27,39]. CTCs are very specific and not found in patients without known primary malignant tumors. Therefore, the ability to use CTCs to predict prognosis and therapeutic efficacy has been investigated by CTC enumeration [25]. Quantification of CTCs in patients with metastatic breast cancer showed that patients with five or more CTCs per unit volume of blood at baseline had a median decrease in progression-free survival and overall survival compared to those with fewer than five CTCs [49]. Another clinical trial indicated that patients with CRC in whom over 3 CTCs per 7.5 mL blood were detected had shorter median and overall survival compared to patients with fewer than 3 CTCs, and these differences persisted at follow-up time points after therapy [50]. These results demonstrate that prognosis can be predicted by only analyzing the number of CTCs in the blood. Of course, many clinical studies have enumerated CTCs in various solid cancers including lung cancer and pancreatic cancer to predict prognosis as well as in breast and colon cancer [51,52]. As these significant results in clinical trials are continually compiled, CTC enumeration may be used to guide standard treatment for cancer patients in the clinic [25]. Furthermore, advanced clinical trials of CTCs analysis are currently evaluating the possibility of chemotherapies for patients with cancer who show drug resistance. A proof of principle study of breast cancer patients showed an objective response of human epithelial growth factor receptor (*HER)*-2-negative tumor patients to *HER*-2 gene amplification on CTCs followed by administration of trastuzumab [49]. In this trial, early breast cancer patients with *HER*-2-negative (tissue in primary site) and cytokeratin-19 (*CK19*) mRNA-positive CTCs were randomly assigned and administered trastuzumab [49]. A decrease in the number of CTCs and significant increase in disease-free survival rate were observed in *HER*-2-negative patients following treatment with trastuzumab [49]. This clinical trial demonstrated that CTCs can be analyzed and targeted to overcome drug resistance and enhance therapeutic efficacy. Furthermore, the prognosis for anticancer drugs used for different types of solid tumors can be predicted. For example, CTC analysis in mutation of epithelial growth factor receptor can be utilized to predict the prognosis of chemotherapies, such as lapatinib or gefitinib, which targets this receptor, for lung cancer treatment [50,52]. Nevertheless, the criteria for the scope of the preliminary analysis and analysis parameters for evaluating circulating biomarkers remains unclear [39]. Therefore, additional clinical trials are needed to establish standardized methods for the diagnoses, prognosis prediction, and treatment using CTC analysis.

## 5. Limitation of Circulating Tumor Cells (CTCs) Analysis for Clinical Applications

As described above, despite the numerous clinical studies related to CTC analysis in cancer patients, these analyses are not routinely used as cancer therapy by physicians. This may be because of the large number of methods for detecting CTCs by physicians and biologists, making it difficult to choose the optimal method [53]. There is a lack of consensus regarding technical approaches to various aspects of the methodology, such as the desired sample type, storage conditions, candidate molecules, and appropriate detection techniques [53]. Even if the detection technique is refined, CTC populations with a non-epithelial phenotype are not detected because CTC detection is based on an epithelial marker [38]. Heterogeneity of CTCs is to be a cause of differentiation from the genetic character of the primary site of cancer and may lower the success rate of diagnosis and prognosis prediction [53,54]. Through large-scale research, these issues can be resolved and increase the usefulness of CTCs as biomarkers [53,54]. Therefore, the development of routine methods and standard biomarkers based on CTCs would be beneficial for treating many cancer patients and improving clinical outcomes.

## 6. Conclusions

Recently, CTCs have been widely analyzed as an alternative for diagnosis, predicting prognosis, and establishing treatment strategies. However, a very complicated process is required to detect and analyze CTCs; therefore, actual clinical applications are limited. Challenges such as standardizing biomarkers that can be applied to each solid cancer or distinguishing resistance even in the same type of cancer must be overcome. However, technological advances are currently being accomplished and large-scale clinical studies are being attempted. Because the unique information that can be acquired by CTC analysis has great potential to understand the peculiar environments of solid tumors, future applications of CTC analysis in the clinic would be a very useful technology.
